# Modeling the Photocatalytic Mineralization in Water of Commercial Formulation of Estrogens 17-β Estradiol (E2) and Nomegestrol Acetate in Contraceptive Pills in a Solar Powered Compound Parabolic Collector

**DOI:** 10.3390/molecules200713354

**Published:** 2015-07-22

**Authors:** José Colina-Márquez, Fiderman Machuca-Martínez, Gianluca Li Puma

**Affiliations:** 1 Program of Chemical Engineering, Universidad de Cartagena Piedra de Bolívar Campus, Av. El Consulado 48-152, A.A. 130015, Cartagena de Indias, Colombia; 2 School of Chemical Engineering, Universidad del Valle, Campus Meléndez, Calle 13 No. 100-00, A.A. 25360 Cali, Colombia; E-Mail: fiderman.machuca@correounivalle.edu.co; 3 Environmental Nanocatalysis & Photoreaction Engineering, Department of Chemical Engineering, Loughborough University, Loughborough LE11 3TU, UK; E-Mail: g.lipuma@lboro.ac.uk

**Keywords:** emerging contaminants, pharmaceuticals, degradation, photoreactor design, six-flux model, UV-photolysis, Langmuir–Hinshelwood, modeling and simulation

## Abstract

Endocrine disruptors in water are contaminants of emerging concern due to the potential risks they pose to the environment and to the aquatic ecosystems. In this study, a solar photocatalytic treatment process in a pilot-scale compound parabolic collector (CPC) was used to remove commercial estradiol formulations (17-β estradiol and nomegestrol acetate) from water. Photolysis alone degraded up to 50% of estradiol and removed 11% of the total organic carbon (TOC). In contrast, solar photocatalysis degraded up to 57% of estrogens and the TOC removal was 31%, with 0.6 g/L of catalyst load (TiO_2_ Aeroxide P-25) and 213.6 ppm of TOC as initial concentration of the commercial estradiols formulation. The adsorption of estrogens over the catalyst was insignificant and was modeled by the Langmuir isotherm. The TOC removal via photocatalysis in the photoreactor was modeled considering the reactor fluid-dynamics, the radiation field, the estrogens mass balance, and a modified Langmuir–Hinshelwood rate law, that was expressed in terms of the rate of photon adsorption. The optimum removal of the estrogens and TOC was achieved at a catalyst concentration of 0.4 g/L in 29 mm diameter tubular CPC reactors which approached the optimum catalyst concentration and optical thickness determined from the modeling of the absorption of solar radiation in the CPC, by the six-flux absorption-scattering model (SFM).

## 1. Introduction

Pharmaceuticals and metabolites residues in the aquatic environment are cause of concern to many agencies, institutions and governments worldwide. Actions for monitoring their occurrence, preventive measures and novel technologies for their containment are currently being evaluated at national and international level [1,2,3,4,5,6]. Among many pharmaceuticals, endocrine disruptors in water are contaminants of emerging concern due to the risk they pose to the aquatic ecosystems and to the environment. Compounds with estrogenic, progestagenic and/or androgenic activities can have significant effect on humans and wildlife [7,8,9,10]. For example, the disruptive impact of 17-α ethynylestradiol (EE2) to the fish population in an experimental lake was demonstrated in a seven-year study, which showed near extinction of fish after four years of EE2 dosing, due to reproductive failure [11]. The female contraceptive pill active compounds 17-β estradiol (E2) and nomegestrol have recently been formulated as an alternative to pills containing the more common synthetic estrogen EE2, since these hormones are structurally identical to endogenous estrogen in women [12].

Current municipal wastewater treatment plants are unable to completely remove or destroy pharmaceuticals from domestic wastewater [13,14]. Advanced oxidation processes (AOPs), which are based on the generation of highly powerful reactive oxidative species (e.g., hydroxyl radicals), have been proposed as alternative processes to inactivate the biological and physiological effect of pharmaceuticals in water. Among AOPs, photocatalytic oxidation over an irradiated semiconductor photocatalyst (often titanium dioxide (TiO_2_)) has proven to be effective in the removal of pharmaceuticals including estrogens [15,16,17,18]. Most studies that investigate the photocatalytic degradation of estrogens deal with idealized systems, using ultrapure water, synthetic chemicals and laboratory reactors [19,20,21,22,23,24]. However, there is a little information on the effectiveness of photocatalysis for the destruction of commercial estrogens formulations at pilot-scale and using real water. These waters may be the effluents from pharmaceutical manufacturing processes.

**Figure 1:**
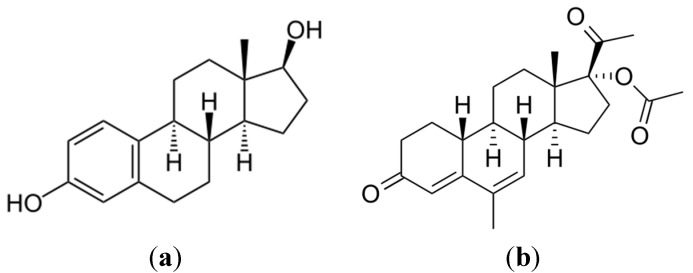
Chemical structures of the estrogens investigated: (**a**) 17-β estradiol and (**b**) Nomegestrol acetate.

In this study, we investigate the treatment of commercial estradiols (17-β estradiol and nomegestrol acetate, Figure 1) aqueous solutions obtained from female contraceptive pills, in a pilot-scale compound parabolic collector (CPC) operated using a solar photocatalytic treatment process and titanium dioxide (TiO_2_) suspensions. The radiation field in the CPC was modeled and the spatial distribution of the volumetric rate of photon absorption (VRPA) was evaluated by applying the six-flux photon absorption-scattering model (SFM) [25,26,27]. This model tracks scattered photons along the six directions of the Cartesian coordinates. The optical parameters of the catalyst suspension in water were averaged across the spectrum of the incident solar light to simplify the modeling methodology. The time-dependent degradation profiles of the commercial estrogens formulation in tap water were determined following the explicit consideration of the volumetric rate of photon absorption in the reaction kinetics and a mass balance across the CPC. The dependence of the treatment of the commercial formulation of estradiols on catalyst concentration and optical thickness was correlated to the rate of photon absorption in the reactor.

## 2. Results and Discussion

### 2.1. Solar Photolysis

**Figure 2:**
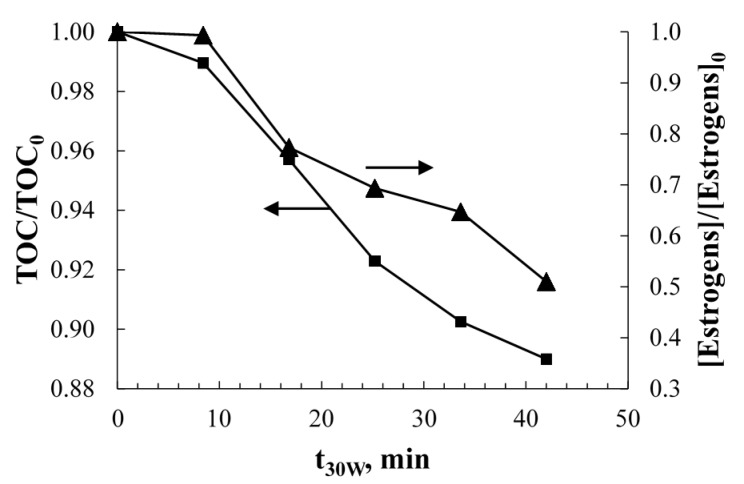
Solar photolytic degradation (**right axis**) and mineralization (**left axis**) of the commercial formulation of E2 and nomegestrol acetate mixture with an initial concentration of 5 ppm of estrogens and 388 ppm of TOC at pH 4.5 and with 97.2 kJ/m^2^ of accumulated solar radiation (measured as UV-A).

Figure 2 shows the rate of degradation and mineralization of the commercial estrogen mixture in the CPC by solar photolysis, in the absence of catalyst. The estrogens concentration and TOC removal after 42 min of irradiation and at a photon irradiance of 30 W/m^2^, was 49% and 11%, respectively.

The observed behavior is similar to the rate of photolysis reported in previous studies [23]. The estrogen mixture shows significant degradation under UV radiation exposure, especially with UV-A and UV-C radiation [23]. Nonetheless, the TOC removal was not as fast as the removal of estrogens. This may result from the presence of excipients compounds in the commercial estradiols formulation, consisting of lactose monohydrate, microcrystalline cellulose, magnesium stearate and polyvinyl alcohol, which are not easily mineralized by UV photolysis. In addition, the transformation products by photolysis are expected to degrade at slower rates, since the aliphatic derivatives of the photolytic degradation of estrogens can be more stable than the aromatic rings under UV irradiation conditions [28]. Although solar radiation only contains a small proportion of UV-C, the collective contribution of the other UV components can be sufficient for breaking chemical bonds in the estrogens molecules [29]. The effect of solar photolysis is usually significant for the degradation of the estrogens parent compound in natural waters. However, in heterogeneous photocatalysis, the absorption of UV photons by the photocatalyst, in well designed reactors, is several orders of magnitude higher than the absorption of photons by the molecules in solution [23], therefore the effect of photolysis can often be neglected when the contaminants and TOC removals are modeled.

### 2.2. Adsorption of Contaminants on the TiO_2_ Catalyst Surface

**Figure 3:**
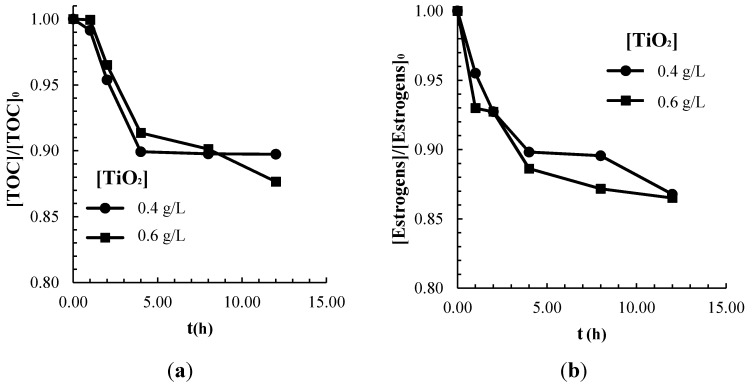
Adsorption of commercial estradiol at 0.4 and 0.6 g/L of catalyst loading with an initial concentration of 7 ppm of estrogens and 429 ppm of TOC: (**a**) TOC removal and (**b**) Estrogens removal.

The results of adsorption of the commercial estrogens mixture onto TiO_2_ are presented in Figure 3 in terms of residual estrogens and TOC concentrations. The catalyst loadings selected in this study were limited to those concentrations that maximized the rate of photon absorption in the CPC and that yielded the fastest rate of contaminants degradation. After 12 h of stirring, under dark conditions at pH 4.5, approximately 13.5% of the initial estrogens concentration was adsorbed onto the surface of TiO_2_. The role of adsorption can be significant in photocatalysis, since it can be rate-limiting to the contaminant degradation kinetics. The adsorption of hydroxyl anions onto the TiO_2_ surface promotes the generation of free hydroxyl radicals via electron exchange, which in turn oxidize water contaminants [30,31]. However, hydroxyl anions and the contaminant molecules compete for adsorption on the catalyst surface sites, therefore, if the fractional coverage of OH is lower than that of estrogens and drug additives, this might have an adverse effect on the rate of generation of oxidative species. Contrasting to this effect, if the adsorption of the chemical species of interest is weak, the observed photocatalytic degradation kinetics of the pollutant may be affected negatively and controlled by the rate of mass transfer of contaminants from the bulk to the catalyst surface.

**Figure 4:**
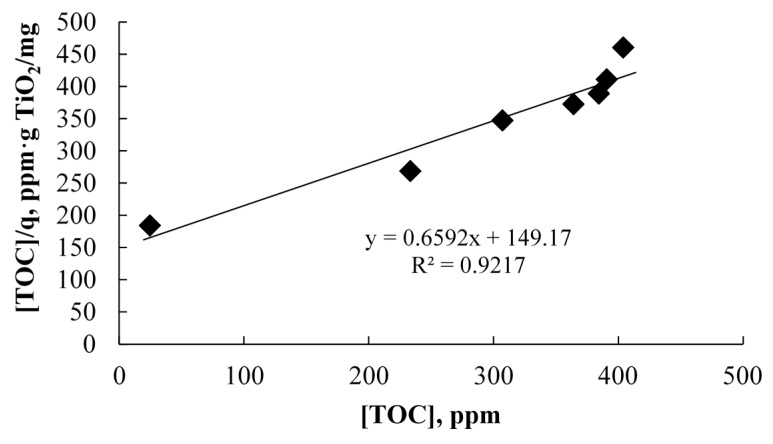
Linear regression of the Langmuir isotherm obtained from experiments at 30 °C, pH 6.9 and with 0.6 g/L of catalyst load.

The TOC of the estrogenic commercial drug was considered for modeling the adsorption phenomena onto the TiO_2_ photocatalyst. The observed adsorption of estrogens in terms of TOC removal shows a typical monolayer behavior, which can be described by a Langmuir isotherm, according to the adsorption equation:

(1) $$q=\frac{q_{0}K_{ads}[TOC]}{1+K_{ads}[TOC]}$$

where *q* is the amount of adsorbate per amount of adsorbent (mg_(TOC)_/g TiO_2_), *q*_0_ is the maximum amount of TOC adsorbed, [TOC] is the concentration of estrogens in solution (mg_(TOC)_/L) and *K_ads_* is the adsorption equilibrium constant (L/mg_(TOC)_). After rearrangement Equation (1), can be written as a linear expression, Equation (2), which allows the estimation of the adsorption parameters by linear fitting of the TOC adsorption results (Figure 4).

(2) $$\frac{\left[TOC\right]}{q}=\frac{1}{q_{0}K_{ads}}+\frac{1}{q_{0}}[TOC]$$

The corresponding *q*_0_ was 1.52 mg_(TOC)_/g TiO_2_, whereas *K_ads_* was 4.42 × 10^−3^ ppm^−1^. The relatively small value of the equilibrium constant, *K_ads_*, suggest weak estrogens adsorption and pseudo first-order adsorption kinetics, as also shown in Figure 3. It is also possible that multilayer adsorption can be relevant at higher substrate concentrations above 400 ppm TOC since the Langmuir isotherm appears to deviate from the experimental data.

#### 2.2.1. Photocatalytic Oxidation of a Commercial Formulation of Estrogens

**Figure 5:**
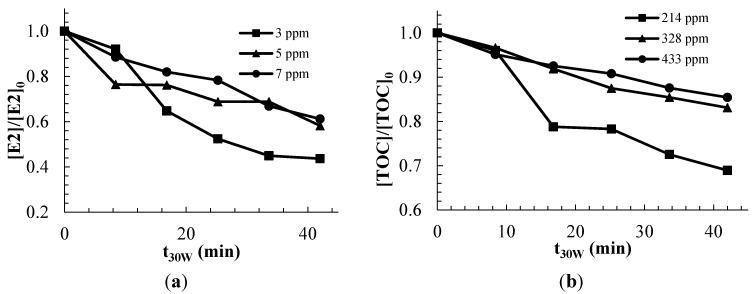
Effect of the initial concentration of the contaminant on the degradation rate with a catalyst load of 0.6 g/L; (**a**) Estrogens degradation; (**b**) TOC removal.

The experiments performed in the pilot scale photoreactor, in the presence of suspended TiO_2_, shows that the overall degradation rate of the commercial estrogens mixture increased as the initial concentration of the hormone disruptor decreased (Figure 5).

The estrogens removal was 56.7%, 41.7% and 38.7% for 3, 5 and 7 ppm of initial estrogens concentration, respectively, which means that the removal rates (ppm/min) increased as the initial estrogen concentration increased, whereas the TOC removal was 31.1%, 16.9% and 14.6% for 214, 328 and 433 ppm of initial TOC concentration, respectively. This behavior is consistent with the prevalent trend observed in other studies involving the photocatalytic oxidation of contaminants in water [23,32,33,34]. In heterogeneous photocatalysis, the apparent contaminants oxidation rates can be limited by adsorption, therefore, the initial concentration of contaminants can have a significant effect on the observed degradation rate. According to the Langmuir kinetic model, lower initial concentrations lead to first-order rate law, whereas higher concentrations lead to zero-order rate law. Figure 5 shows faster removal of both estrogens and TOC at the lowest initial concentrations, which is consistent with a pseudo first-order behavior.

It should be observed that the rates of degradation of estrogens in the presence of TiO_2_ (Figure 5a) are not too dissimilar to the rate observed in the absence of catalyst (Figure 2), which contrast with other studies that have shown faster estrogens removal in the presence of TiO_2_ photocatalyst [23]. This apparent coincidence may erroneously suggest that photolysis alone may be the most important factor responsible for the degradation of the contaminants in the aqueous solutions. However, it should be observed that at the same time, the absorption of UV photons by the photocatalyst is several orders of magnitude higher than the absorption of photons by the molecules in solution [23], therefore, the effective photon irradiance available for the photolysis of the molecules in solution is also several orders of magnitude smaller in comparison to the case in which the photocatalyst is absent. Since the rate of contaminants photolysis is first-order on the photon irradiance (Beer–Lambert law), it can be concluded that the contribution of photolysis alone should be insignificant in the observed degradation and mineralization of the contaminants in the presence of a photocatalyst. This conclusion is further supported by the analysis of the transformation products of estrogens (EE2) observed under UVA photocatalysis, which show that oxidation of estrogens occurs by hydroxyl radical attack [19,35]. The mineralization results (Figure 5b) show faster TOC removal in the presence of TiO_2_ in comparison to photolysis alone (Figure 2).

#### 2.2.2. Modeling and Experimental Validation of the Solar Photocatalytic Degradation of Contraceptive Pills Formulations

Due to the complex nature of the commercial contraceptive pill formulation, the photocatalytic treatment process was followed and modeled in terms of TOC removal.

The modified Langmuir–Hinshelwood (L–H) rate model proposed by Li Puma *et al.* [27], was adopted to describe the photocatalytic degradation of the commercial estradiol mixture. The radiation field in the CPC was analyzed using the six-flux absorption scattering model (SFM) [26]. The set of equations are summarized as follows:

(1) Reactor mass balance (expressed as differential equation in polar coordinates):
  (3) $$\frac{d{\left[TOC\right]}_{r,\text{θ}}}{dt_{30W}}=r_{TOC}$$
(2) Contaminant rate law:
  (4) $$r_{TOC}=-k_{T}\frac{K_{R}[TOC]}{1+K_{R}[TOC]}\int {\left(LVRPA\right)}^{m}dV_{R}$$
(3) Mass balance in the batch recirculation system:
  (5) $${\left[TOC\right]}_{i+1}^{in}=\frac{{\left[TOC\right]}_{i}^{in}(V_{T}-V_{R})+{\left[TOC\right]}_{i}^{out}V_{R}}{V_{T}}$$
(4) Hydrodynamic model for turbulent flow in the CPC, which was operated in the turbulent flow regime:
  (6) $$\frac{v_{z}}{v_{z,max}}={\left(1-\frac{r}{R}\right)}^{\frac{1}{n}}$$
  (7) $$n=0.41\sqrt{\frac{8}{f}}$$
  (8) $$v_{z,average}=\frac{Q}{\pi r^{2}}$$
  (9) $$\frac{v_{z,\max}}{v_{z,average}}=\frac{\left(n+1)(2n+1\right)}{2n^{2}}$$
(5) Optical properties of the catalyst and SFM parameters:
  (10) $$\text{ω}=\frac{\text{σ}}{\text{σ}+k}$$
  (11) $$a=1-\text{ω}p_{f}-\frac{4{\text{ω}}^{2}p_{s}^{2}}{1-\text{ω}p_{f}-\text{ω}p_{b}-2\text{ω}p_{s}}$$
  (12) $$b=\text{ω}p_{b}-\frac{4{\text{ω}}^{2}p_{s}^{2}}{1-\text{ω}p_{f}-\text{ω}p_{b}-2\text{ω}p_{s}}$$
  (13) $${\text{ω}}_{corr}=\frac{a}{b}$$
  (14) $$\text{t}=(\text{σ}+\text{κ})\text{δ}C_{cat}$$
  (15) $${\text{τ}}_{app}=a\text{τ}\sqrt{1-{\text{ω}}_{corr}^{2}}$$
  (16) $$\text{γ}=\frac{1-\sqrt{1-{\text{ω}}_{corr}^{2}}}{1+\sqrt{1-{\text{ω}}_{corr}^{2}}}e^{-2{\text{τ}}_{app}}$$
  (17) $${\text{λ}}_{{\text{ω}}_{corr}}=\frac{1}{a(\text{σ}+\text{κ})C_{cat}\sqrt{1-{\text{ω}}_{corr}^{2}}}$$
(6) The local volumetric rate of photon absorption (LVRPA) calculated from the SFM:
  (18) $$LVRPA=\frac{I_{0}}{{\text{λ}}_{\text{ω}corr}{\text{ω}}_{corr}(1-\gamma )}[({\text{ω}}_{corr}-1+\sqrt{1-{\text{ω}}_{corr}^{2}})e^{-\frac{r_{p}}{{\text{λ}}_{\text{ω}corr}}}+\gamma ({\text{ω}}_{corr}-1+\sqrt{1-{\text{ω}}_{corr}^{2}})e^{\frac{r_{p}}{{\text{λ}}_{\omega corr}}}]$$

The parameters were fitted from the experimental results obtained with 0.6 g/L of TiO_2_ using the optical properties of the Aeroxide P-25, shown in Table 1 [36].

**Table 1:** Optical parameters of TiO_2_ Aeroxide P-25 in water averaged across the solar radiation spectrum up to the maximum wavelength that can photoactivate TiO_2_ (λ = 385 nm) [36].

Parameter	Value
Absorption coefficient (κ)	174.7 m^2^/kg
Extinction coefficient (β)	1470.5 m^2^/kg
Scattering coefficient (σ)	1295.8 m^2^/kg
Forward scattering probability (*p_f_*)	0.110
Backward scattering probability (*p_b_*)	0.710
Side scattering probability (*p_s_*)	0.045

The scattering albedo ω, calculated from Equation (10), was 0.88, and the corrected albedo, ω*_corr_*, was 0.75, which was estimated from Equations (10)–(13).

**Figure 6:**
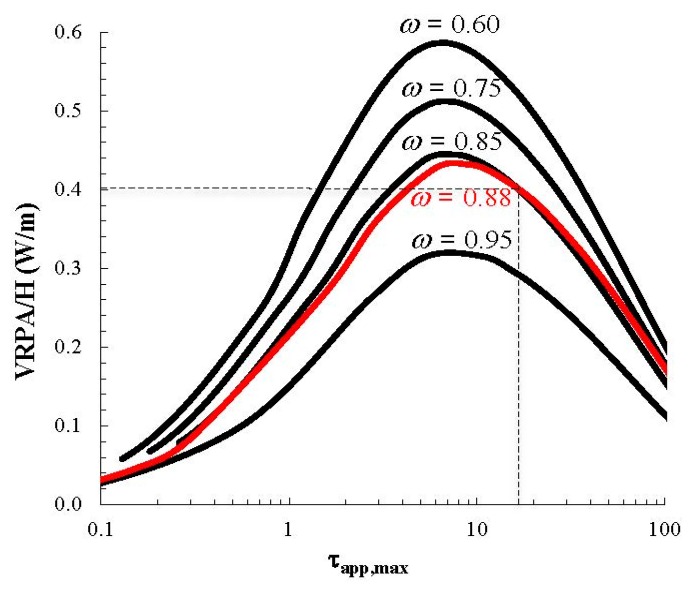
Effect of the maximum optical thickness on the VRPA per unit of length of a CPC solar reactor. Adapted from Colina-Marquez *et al*. [37].

**Figure 7:**
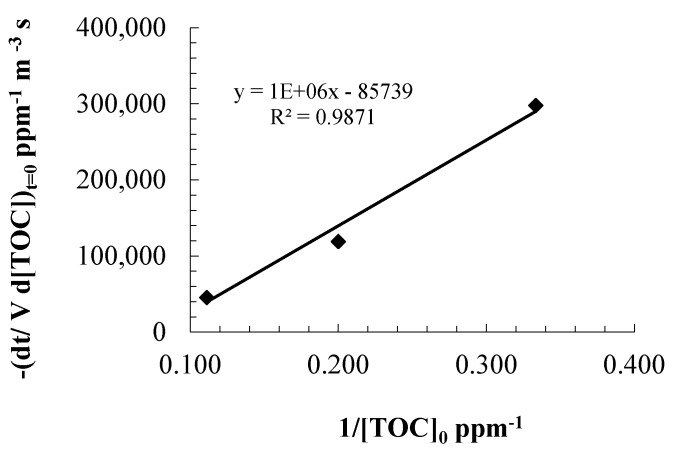
Linear regression for fitting parameters of the kinetic model.

The optical thickness (τ (Equation (14)) in the CPC reactor, estimated for a TiO_2_ catalyst loading of 0.6 g/L and a reactor diameter of 33 mm, was equal to 29.0, whereas the apparent maximum optical thickness τ*_app_*_,*max*_ from Equation (15), at this catalyst concentration, was equal to 17.1. The volumetric rate of photon absorption per unit length of the solar CPC reactor (*VRPA*/*H*) could then be estimated using the modeling results of the CPC solar reactor previously reported [37], shown in Figure 6. These results were calculated for the solar irradiation conditions of Cali (Colombia), which, however, were very similar to the prevalent irradiation conditions of this study (latitude 3.5°). At τ*_app_*_,*max*_ = 17.1, the *VRPA/H* for ω = 0.88 equals 0.405 W/m. Since the total length of the CPC reactor used in the experiments was 12 m, the corresponding *VRPA* was 4.86 W. Combining Equations (3) and (4) and inverting yields Equation (19),

(19) $$\frac{1}{\left(-{\text{V}}_{T}\frac{d[TOC]}{dt_{30W}}\right)}=\frac{1}{K_{R}K_{T}{\left(VRPA\right)}^{0.5}}(\frac{1}{\left[TOC\right]})+\frac{1}{K_{T}{\left(VRPA\right)}^{0.5}}$$

and the kinetic parameters *k_T_* and *K_R_* were calculated by performing a linear fitting of the experimental data, with the TOC removal rates determined at time zero (Figure 7). The dimensionless parameter *m* in Equation (4) is related to the probability of electron–hole recombination and can take values within the 0.5–1.0 range, however, when the UV irradiance is significant and is not the limiting step of the photocatalytic reaction, *m* can be fixed as 0.5 [38].

From the slope and the intercept with the vertical axis (Equations (20) and (21)), the reaction kinetics and adsorption constants were estimated:

(20) $$slope=\frac{1}{K_{R}K_{T}{\left(VRPA\right)}^{0.5}}=348,947$$

(21) $$\mathrm{int}ercept=\frac{1}{K_{T}{\left(VRPA\right)}^{0.5}}=25,773$$

The estimated kinetic constants values were *k_T_* = 1.76 × 10^−5^ ppm·m^1.5^/s·W^0.5^ and *K_R_* = 7.386 × 10^−2^ ppm^−1^. The adsorption constant *K_ads_* estimated with the modified L–H kinetic model was one order of magnitude greater than the equilibrium adsorption constant *K_ads_*, determined from the dark adsorption experiments. This apparent discrepancy results from the modification of the physical and chemical properties of the catalyst surface when this is irradiated with UV photons and when electron-hole pairs are generated [39]. When the TiO_2_ surface is irradiated, there is an electron exchange with the hydroxyl anions with pH decrease, therefore, the net electric charge of the catalyst surface becomes positive and this effect might favor the physical adsorption of the anionic chemical species from the contaminants in solution. Nonetheless, the adsorption constant *K_R_* estimated still suggests a weak adsorption of the contaminants.

The degradation of the commercial estradiol formulation was modeled in terms of TOC removal, using the proposed model and the kinetic parameters determined for the commercial estradiol formulation. Since the experimental pilot-scale CPC reactor was a flow-through reactor with external recirculation, the simulations and the latter validation were carried out considering the concentration changes after multiple passes through the solar photoreactor. The total number of passes in each simulation was estimated with Equation (22):

(22) $$n_{pass}=\frac{Qt_{30W}}{V_{R}}$$

where *t*_30*W*_ corresponds to the total time of the simulations. One hundred small sub-reactors (*j* = 1 to 100) of equal length (*L*_j_ = *L*/100) were considered to model the flow-through the CPC reactor during each pass, and the calculations were made considering that each sub-reactor behaved as a turbulent flow reactor. Considering the velocity profiles trough the cross-area of the reactor and the rate law (Equation (4)), the mass balance equation was expressed as follows:

(23) $$Q\frac{d{\left[TOC\right]}_{r,\theta }}{dV_{R}}=-\frac{K_{T}K_{R}{\left[TOC\right]}_{r,\theta }}{\left(1+K_{R}\;{\left[TOC\right]}_{j}^{in}\right)}{\left(LVRPA\right)}_{r,\theta }^{m}$$

where ${TOC}_{j}^{in}$ is the feed TOC concentration to each sub-reactor. Since the LVRPA varies in both the radial and the angular directions, the local reaction rate also varies across the cross sectional area of the tube. The TOC concentration in the reactor further changes along the *z*-axis, therefore, the initial condition was set as follows:

(24) $$z=0,\;TOC_{j=1}^{in}=TOC_{i+1}^{in}$$

where ${\left[TOC\right]}_{i+1}^{in}$ is the TOC concentration from the overall mass balance after each pass (Equation (5)). Obviously, for the first pass ${\left[TOC\right]}_{i=1}^{in}$ equals the initial TOC concentration in the reactor feed stream.

The local TOC concentration at the exit cross section of each sub-reactor was estimated for each point of the polar grid by solving the differential equation (Equation (23)):

(25) $${\left[TOC\right]}_{r,\theta }^{out}=exp[ln({\left[TOC\right]}_{j}^{in})-\frac{k_{R}k_{T}}{v_{Z}(1+K_{R}{\left[TOC\right]}_{j}^{in})}\int_{0}^{L_{j}}{\left(LVRPA\right)}_{r,\theta }^{m}dz]$$

where *v_z_* is the average velocity of the fluid, which is a function of the radial coordinate (Equation (6)). The average TOC concentration at the exit of each sub-reactor was calculated by integrating the TOC concentration profile along the radial and angular directions taking into account the liquid flow rate:

(26) $${\left[TOC\right]}_{j}^{out}=\frac{\int_{0}^{2\pi }\int_{0}^{R}rv_{z}{\left[TOC\right]}_{r,\theta }^{out}drd\theta }{Q}$$

which is equivalent to mixing the fluid at the exit of each sub-reactor.

Finally, Equations (25) and (26) are solved iteratively for each sub-reactor after setting the initial condition:

(27) $${\left[TOC\right]}_{j+1}^{in}={\left[TOC\right]}_{j}^{out}$$

until the reactor exit at z = *L* is reached (*j* = 100).

The reactor analysis presented above neglects the axial mixing of the fluid. Under turbulent flow conditions, it was necessary to assume complete radial and angular mixing, resembling the behavior of a continuous-stirred tank reactor (CSTR) across each cross section.

The calculation of the TOC concentration was made for each pass through the reactor until the number of passes established in Equation (22) was completed (*i* =1 to *n_pass_*).

**Figure 8:**
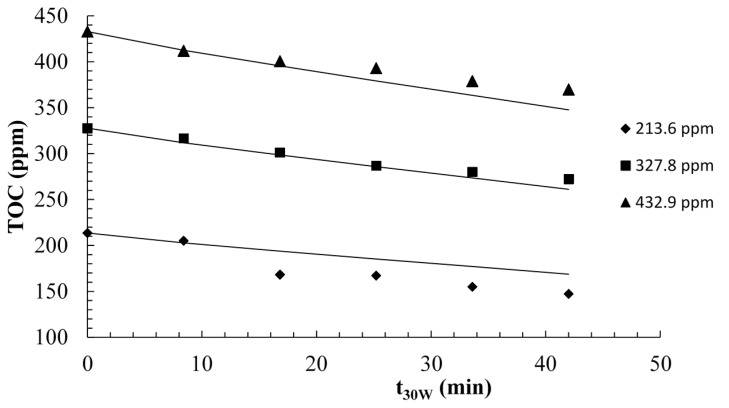
Modeling of the photocatalytic mineralization of commercial estradiol. The initial pH was 4.6 and the catalyst loading was 0.6 g/L.

The solid lines illustrated in Figure 8 correspond to the data generated by the model with the kinetic parameters *k_T_* and *K_R_* determined. The model described the experimental data satisfactorily, although it slightly overestimated the concentration profile at *t*_30*W*_ > 15 min, when the initial TOC concentration was 213.6 ppm. At lower TOC concentration, the catalyst surface may not be fully saturated with the substrates, and as a result a higher fraction of water adsorption yielded a higher rate of hydroxyl radical generation, ultimately contributing to a higher overall removal of the substrate. Whereas for the higher concentrations, the observed behavior could resemble to zero-order kinetics, for lower concentrations this behavior was closer to a first-order kinetics.

**Figure 9:**
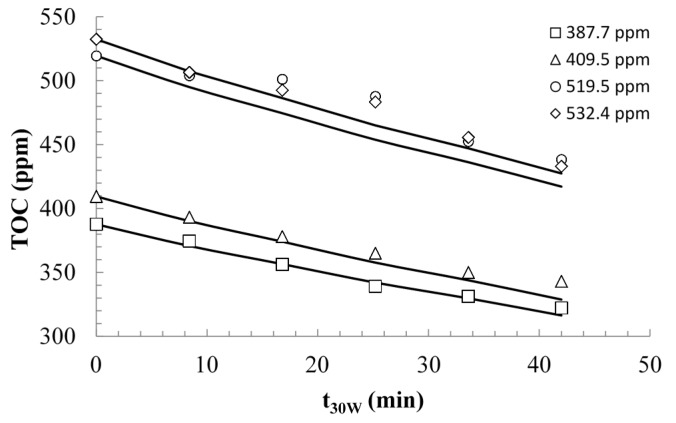
Validation of the TOC photocatalytic degradation with 0.4 g/L of catalyst load.

The photoreactor model was further validated by comparing the experimental and model results of the the mineralization of the commercial estradiol formulation, using a different catalyst loading (0.4 g/L) and four initial TOC concentrations. The value of the *VRPA*/*H* for the model simulations was determined from Figure 6, using the new value of the apparent maximum optical thickness (τ*_app_*_,*max*_), since this parameter is a function of the catalyst loading. Using Equation (15) τ*_app_*_,*max*_ was equal to 11.43, and the *VRPA* was 5.18 W, which is slightly greater than the value corresponding to the catalyst loading of 0.6 g/L. In consequence, a greater TOC removal using 0.4 g/L of catalyst was expected, since the new *τ_app_*_,*max*_ approaches the optimum value (Figure 6). The results shown in Figure 9 demonstrate that the model described the photocatalytic degradation of estrogens satisfactorily, for both 387.7 and 409.5 ppm of TOC as initial concentrations.

The model appeared to underestimate the experimental results at higher TOC concentrations. One explanation for this behavior is that the underlying assumption of monolayer adsorption described by the L–H modeling approach may begin to fail, and that at these high TOC concentrations, the adsorption phenomena may be of multilayer nature, with complete saturation of the catalyst surface. The results shown in Figure 4 also suggest that a multilayer coverage of the catalyst at TOC concentrations higher than 400 ppm may be approached. Both cases lead to a decrease of the rate of •OH radical generation due to a higher fractional coverage of the surface with estradiols and, in consequence, to a reduction of the rate of TOC mineralization.

**Figure 10:**
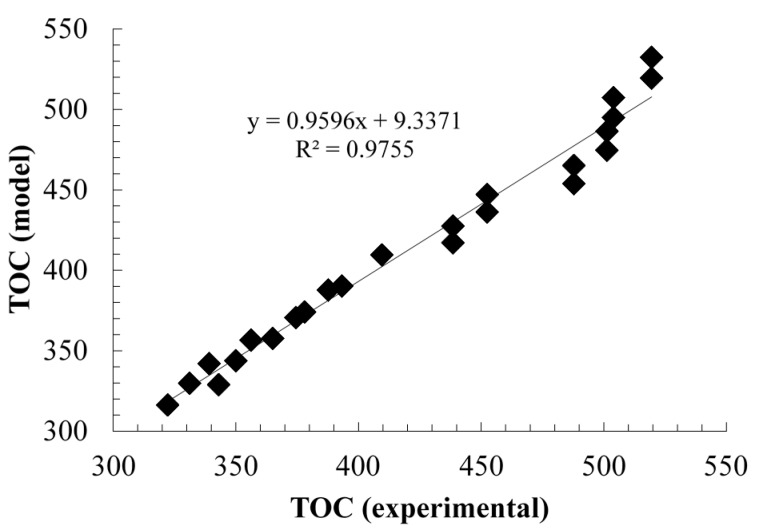
Comparison of the TOC values obtained by the model and the experimental TOC data.

Figure 10 shows the fitting of the TOC values predicted by the model with the experimental data obtained in the validation tests. The R^2^ value points to a satisfactory fitting in general, with low dispersion of the data. As shown in Figure 10, the deviation becomes more significant at higher concentrations of TOC, depicting the possible multilayer adsorption of substrates.

The effect of the catalyst loading is evidenced by the increase of the TOC removal, which was slightly greater with 0.4 g/L in comparison to the results with 0.6 g/L (21.6% *vs.* 19.7%, respectively, for the case of 400 ppm of initial TOC concentration). Our previous study [37] established an optimal catalyst loading of 0.33 g/L for the CPC reactor under the same operating conditions and optical parameters used in this study. With a catalyst loading of 0.4 g/L, which is closer to this optimum, the model predicted a higher rate of contaminant mineralization than with 0.6 g/L, as predicted by the greater value of the *VRPA*. The existence of an optimum in the volumetric rate of photon absorption per unit reactor length (*VRPA*/*H*) is explained by a lower rate of photon adsorption at low catalyst concentrations when the catalyst surface area is insufficient for the absorption of the incident photons, and by a high scattering and “clouding” effects at catalyst concentrations much higher than the optimum, since under this situation the absorption of radiation is effective in a cross sectional area which is smaller than the actual physical cross section of the tube.

## 3. Experimental Section

### 3.1. Materials and Methods

**Figure 11:**
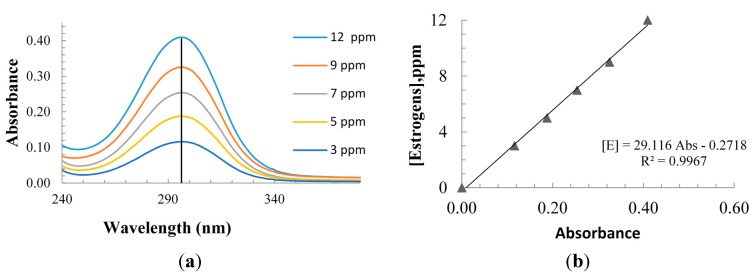
(**a**) UV absorbance of estrogens at different concentrations, showing a peak absorption wavelength at 297 nm. (**b**) Calibration curve for estrogens in tap water.

The commercial contraceptive pill selected for this study contained 1.5 mg of 17-β estradiol (E2, as hemihydrate), 2.5 mg of nomegestrol acetate and excipients compounds (lactose monohydrate, microcrystalline cellulose, magnesium stearate and polyvinyl alcohol of unknown concentration) in each caplet. To prepare the estradiol aqueous solution, the caplets were pulverized and 30 mL of ethanol (Merck^®^) was added to the powdered caplets to extract the estrogens from the powder. The solution was filtered and the extract was lately dissolved in 40 L of tap water to perform the solar experimental tests. The concentration of the estradiols in each experiment was adjusted based on the content of multiple caplets. TiO_2_ Aeroxide^®^ P-25 (Evonik, primary particle size, 20–30 nm by TEM; specific surface area 52 m^2^·g^−1^ by BET; composition 78% anatase and 22% rutile by X-ray diffraction) was used in the experiments. The catalyst was added in the reactor and circulated under dark conditions overnight before exposing the reactor to sunlight the next day. Sampled collected from the reactor containing TiO_2_ were immediately filtered through a 0.45 μm Nylon filter (Millipore, Billerica, MA, USA) prior to immediate further quantitative analysis. The residual concentration of estrogens in the water was determined by UV-spectroscopy (Shimadzu UV1800 spectrophotometer) by measuring the absorbance in the UV region. The calibration curve and the absorbance spectra of estrogens are shown in Figure 11. The total organic carbon (TOC) of the samples was measured using a Shimadzu TOC-VCPH.

The adsorption tests were performed in sealed 500 mL-beakers containing 300 mL of an aqueous solutions of estrogens and suspended TiO_2_ Aeroxide^®^ P-25 at known concentrations. The samples were kept in a chamber under dark conditions and continuous magnetic stirring for 12 h. The concentration of estrogens in the water prior to adding the catalyst and after reaching equilibrium in the presence of catalyst was measured by UV-spectroscopy and TOC analysis.

The incident solar UV radiation accumulated in the photoreactor was measured by a UV radiometer (Delta OHM 210.2) with an UV-B probe, which covers the wavelength range between 280 and 315 nm. The effect of UV-A radiation from the solar radiation spectrum up to the absorption band edge of TiO_2_ (384 nm) was evaluated with a common extrapolation by considering that the UV-B is approximately the 10% of the UV A+B radiation. The dissolved oxygen concentration in the water was monitored with a Spectroquant Pharo 3000. To account for variation in solar irradiance during the day, the photocatalytic treatment time was standardized based on the *t*_30W_ time, which considers that the average UV photon irradiance during a clear sunny day is 30 W/m^2^ [36].

### 3.2. Photocatalytic Reactor

**Figure 12:**
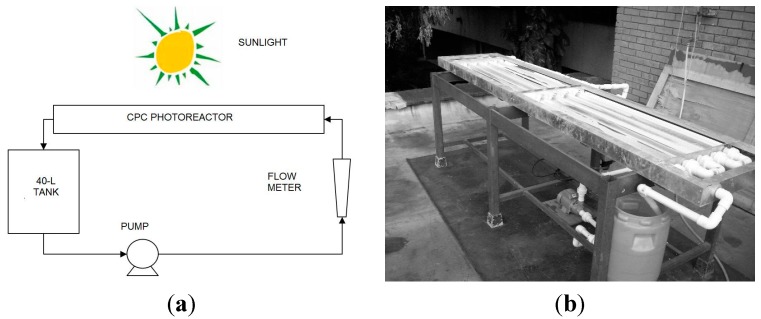
Pilot-scale solar CPC: (**a**) scheme of experimental setup and (**b**) photocatalytic reactor (Universidad del Valle, Cali-Colombia).

The photolytic and photocatalytic tests were performed in a solar CPC photoreactor shown in Figure 12. It consisted of ten Duran^®^ glass tubes (1200 mm in length, 32 mm OD, 1.4 mm wall thickness), supported by a metal structure. The reactor was operated in a recirculation mode using a 40 L recycle feed tank, a recycling centrifugal pump (½ hp of nominal power) that delivered 30.2 L/min. The Reynolds number in the CPC for these operating conditions was 19,400, therefore the flow regime was fully developed and turbulent. The flow rate was measured by a calibrated flow meter. The pipeline and accessories used in the pilot plant were made of PVC, 1-inch diameter.

**Figure 13:**
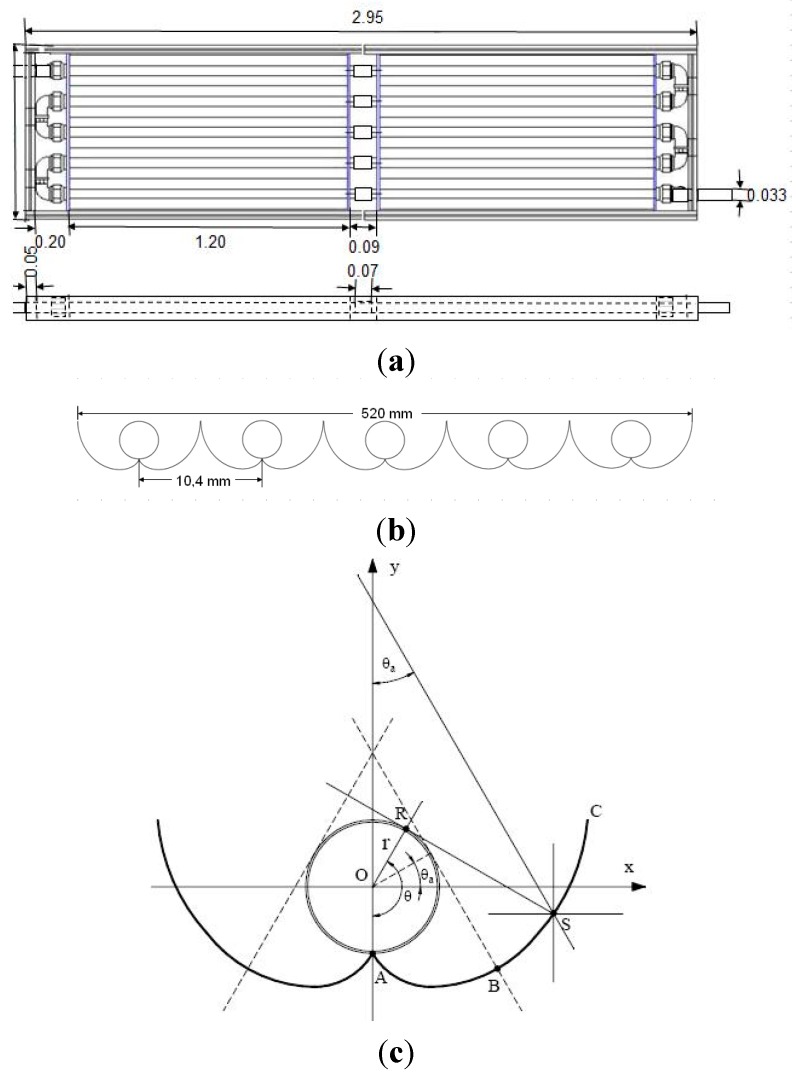
Solar CPC Photoreactor details: (**a**) superior and lateral views and (**b**) frontal view showing the curvature of the compound parabolic collectors; (**c**) CPC involute design.

The geometry of the CPC is described in Figure 13. The curvature of the CPC involute (Figure 13) is described mathematically by the equations:

(28) $$\rho \;=r\theta \;\text{for}\;|\theta |\leq {\text{θ}}_{a}+\text{π}/2\;\text{part AB of the curve}$$

(29) $$\text{ρ}=r\frac{\theta +\theta_{a}+\pi /2-\cos(\theta -\theta_{a})}{1-\sin(\theta -\theta_{a})}\text{ for }\theta_{a}+\pi /2\leq \left|\theta \right|\leq \frac{3\pi }{2}-\theta_{a}\text{ part BC of the curve}$$

where ρ is the radial coordinate of the involute, *r* is the reactor radius, θ is the angular coordinate of the involute and θ*_a_* is the acceptance angle of the collector. For this case, the chosen acceptance angle was 90°.

## 4. Conclusions

The modified Langmuir–Hinshelwood (L–H) rate model, including the effect of photon adsorption and scattering represented by the SFM, modeled satisfactorily the removal of the total organic carbon of commercial estradiols (17-β estradiol and nomegestrol acetate) aqueous solutions, in a solar CPC photoreactor with relative errors of 5%. The application of solar photolysis without catalyst demonstrated a low rate of mineralization, although the rate of estrogens degradation was faster. The effect of solar photolysis could be neglected in the modeling of the TOC removal and the modified L–H rate model accounting for photocatalysis allowed the estimation of the kinetic parameters (reaction kinetics and adsorption constants) independent of the radiant field in the CPC photoreactor. It is important to note that the adsorption constant estimated with the Langmuir isotherm model under dark conditions, was one-order of magnitude greater than the adsorption constant obtained from the modified L–H kinetic model during the photocatalytic experiments. This demonstrates that the adsorption of estrogens over the TiO_2_ catalyst surface is significantly affected under photon irradiation, as a result of the oxidation of the adsorbed estrogens. It was also demonstrated that at catalyst concentrations closer to the optimum predicted from radiation modeling consideration, the rate of TOC removal increased, and that the model could follow these trends. This study highlights the importance of kinetics and reactor modeling, which should always include the effect of the radiation field as a fundamental step, since without photon absorption there cannot be photocatalysis, as well as without reactants, there cannot be reactions. The current literature too often neglects this aspect in kinetic modeling.

## Nomenclature

*a*	SFM parameter, dimensionless
*b*	SFM parameter, dimensionless
*f*	friction factor, dimensionless
*I*	UV radiation intensity, W·m^−2^
*k_T_*	kinetic constant, mol·L^−1^·s^−1^·W−^0.5^·m^1.5^
*K_ads_*	adsorption equilibrium constant, L·mol^−1^
*K_R_*	reaction binding constant, L·mol^−1^
*L*	reactor length, m
*LVRPA*	local volumetric rate of photon absorption, W·m^−3^
*m*	reaction order respect to the LVRPA, dimensionless
*n*	parameter of velocity profile for turbulent regime, dimensionless
*n_pass_*	number of pass of the fluid through the reactor space, dimensionless
*p_b_*	probability of backward scattering, dimensionless
*p_f_*	probability of forward scattering, dimensionless
*p_s_*	probability of side scattering, dimensionless
*Q*	flow rate, m^3^·s^−1^
*r*	radial coordinate, m
*r_p_*	auxiliary coordinate in the photon flux direction, m
*R*	reactor radius, m
*t*	time, s
*t*_30*W*_	standardized 30 W time, s
*TOC*	TOC concentration, mol·L^−1^
*v*	fluid velocity, m·s^−1^
*V*	volume, m^3^
*VRPA*	overall volumetric rate of photon absorption, W·m^−3^
Greek Letters	
δ	reactor thickness, m
γ	SFM parameter, dimensionless
κ	specific mass absorption coefficient, m^2^·kg^−1^
λ	radiation wavelength, nm
θ	polar coordinate, radians
σ	specific mass scattering coefficient, m^2^·kg^−1^
τ	optical thickness, dimensionless
ϖ	declination angle, radians
ω	scattering albedo, dimensionless
Subscripts	
*app*	apparent
*average*	average
*corr*	corrected
*max*	maximum
*min*	minimum
*r*	radial coordinate
*R*	reactor
*total*	total radiation
*T*	total
*z*	axial coordinate
*0*	relative to incident radiation, or initial condition
λ	radiation wavelength
θ	angular coordinate
ω*corr*	corrected albedo
Superscripts	
*in*	reactor inlet
*out*	reactor outlet
